# Fournier's Gangrene Associated with Local Cutaneous HPV Lesions in a Previously Healthy Girl

**DOI:** 10.1155/2013/704532

**Published:** 2013-04-22

**Authors:** Maria Tsinti, Theophani Tsekoura, Evangelos Blevrakis, Ioannis Vlachakis, Amalia Tsilimigaki-Christaki

**Affiliations:** ^1^Department of Paediatrics, Venizelion General Hospital, Knossos Avenue, 71409 Heraklion, Crete, Greece; ^2^Department of Paediatric Surgery, University General Hospital of Heraklion, 71500 Heraklion, Crete, Greece

## Abstract

A case of an 11-year-old prepubertal girl with Fournier's gangrene is presented. The diagnosis was posed after the disease had progressed to the typical clinical picture of frank gangrene. A benign viral infection, common warts, possibly represents the initiating event of the development of this necrotizing soft tissue infection. The development of childhood Fournier's gangrene in association with viral infections has been reported in the past. An optimal outcome was achieved by aggressive antibiotic and surgical treatment despite the late diagnosis. In children, Fournier's gangrene develops in previously healthy individuals with benign diseases often representing the initiating events; thus, high index of suspicion is necessary for an early diagnosis that will lead to the optimal outcome.

## 1. Introduction

Fournier's gangrene (FG) is a potentially fatal necrotizing soft tissue infection of the perineal region. It is defined as a necrotizing fasciitis, of the perineal, perianal, and genital areas with extensive fascial necrosis, obliterative arteritis, and involvement of the adjacent skin. Necrosis of the superficial fascia is always more extensive than that indicated by the extension of skin necrosis. This polymicrobial synergistic infection spreads rapidly along the fascial planes and has a fulminant clinical course [1]. 

FG, initially described as a disease of young males, is now known to affect both sexes and all ages; the greatest incidence is observed among middle-aged immunocompromised men [2]. It represents a very rare clinical entity in childhood, and the majority of cases have been described in infants [3]. The prevalence of FG in the pediatric population remains unknown. Nevertheless, the incidence of necrotizing fasciitis of various anatomic localizations, including the perineal, has been reported to be 0.08/100.000 in children in comparison to 0.4/100.000 in adults [3]. In children, FG appears to have a more benign course and a better outcome compared to adults [4, 5]. Most pediatric cases of FG have been reported in previously healthy children, who may appear well in the early course of the disease and, thus, may escape diagnosis [1, 5]. Predisposing factors such as local trauma and infections, including viral infections, have been described in childhood FG [6]. 

Herein, we report a case of a previously healthy 11-year-old girl with FG. The disease was diagnosed after having had progressed to the typical clinical presentation. The detection of common dermal warts on the vulva and buttocks on clinical examination and the histological findings consistent with HPV infection in the dermal area close to the necrotic lesions strongly suggest that warts represented the initiating event for the development of FG. In addition, we describe this clinical case to emphasize on the meticulous clinical examination and the high index of suspicion, necessary for the early diagnosis and prompt intervention in FG, and on the necessity of aggressive medical and surgical treatment for the optimal outcome. 

## 2. Case Presentation

An 11-year-old, prepubertal, previously healthy girl presented to the pediatric emergency department with a 5-day fever of 40°C and limping. She had presented twice in the pediatric emergency departments with fever on the third and fourth days of the disease without reporting any other complaints and was discharged as the physical examination had been unremarkable. 

**Figure 1:**
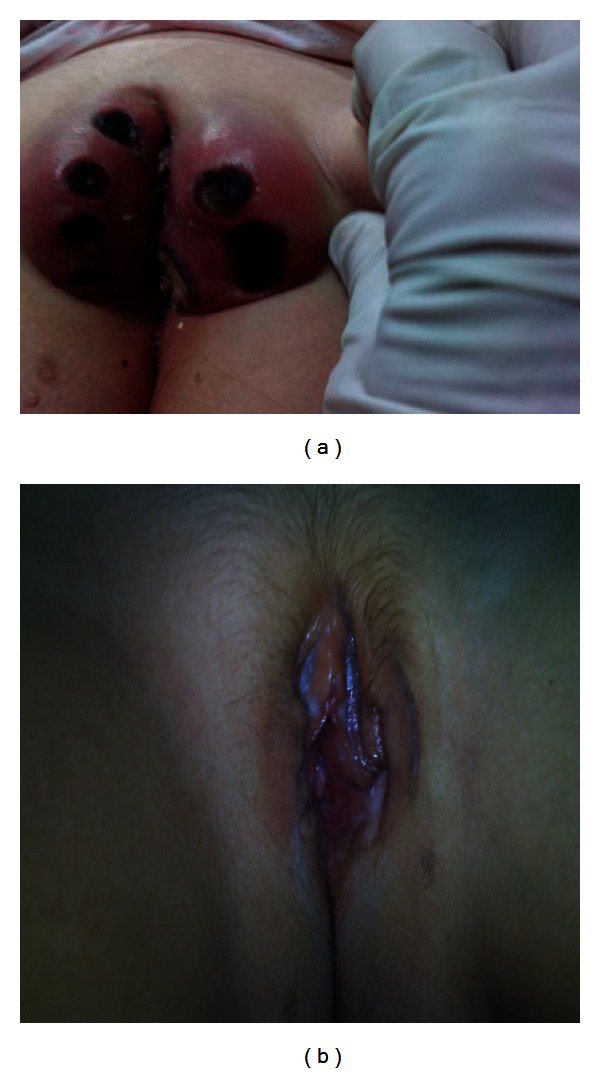
(a) Local necrotic lesions on the labia majora can be observed; the extensive oedema and erythema of the vulva, the black necrotic eschars, covering the ulcers and the purulent exudate. (b) In a ten-month followup, the tissue defect has largely been restored. The intervention of reconstructive surgery is a secondary intension.

On physical examination, she appeared ill and was highly agitated due to severe pain. The evaluation of vital signs revealed temperature of 40°C, heart rate of 120 beats per minute, respiratory rate of 30 breaths per minute, and blood pressure of 100/70 mmHg. The examination of the perineal region revealed severe tenderness, marked vulval edema and erythema, and black ulcerated eschars, of about 1 cm diameter, on the labia majora. The ulcers were well demarcated with irregular edge and malodorous grayish discharge. The labia minora was intact (Figure 1). Bilateral inguinal lymph nodes of 1,5 cm size were palpable but not tender. On dermal examination, common warts of approximately 3 mm size were detected close to the necrotizing lesions on the vulva and the inner surface of the buttocks. No lesions were detected on the labia minora and vagina and meticulous physical examination as well as detailed history excluded the possibility of sexual abuse. The remaining findings of the physical examination were unremarkable. History of previous ulcerative disease was not reported. 

Initial laboratory investigation revealed leukocytosis with white blood cell count of 18600/mm^3^ (75% neutrophils, 4% lymphocytes, and 14% monocytes), elevated infectious markers with erythrocyte sedimentation rate of 60 mm/1 hr and C-reactive protein of 28,5 mg/dL, coagulopathy with prothrombin time of 17,00 sec (control 9,5–14), activated partial thromboplastin time of 41,2 sec (control 25–37), international normalization ratio of 1,41 (control 0,85–1,15), and fibrinogen of 488 mg/dL (control 238–398). Biochemical measurements showed the following concentrations: urea 23 mg/dL, creatinine 0,66 mg/dL, sodium 136 mmol/L, potassium 4,4 mmol/L, glucose 86 mg/dL, ALT 12 U/L, AST 37 U/L, total protein 8,6 g/dL, albumin 4,9 g/dL, CPK 10 U/L, *γ*GT 13 U/L, and LDH 188 U/L. Ultrasonography of the abdomen was normal. Ultrasonography of the perineum revealed a small atractoid suprapubic fluid collection and swelling of the subcutaneous tissue, as well as the presence of small gas bubbles, despite the absence of the clinical sign of crepitus. Bilateral inguinal lymph nodes of 1,5 cm diameter were detected.

On the basis of the clinical picture, the diagnosis of FG was established and was supported by laboratory findings and imaging. Supportive treatment with intravenous fluid administration, transfusion with fresh frozen plasma, and analgesia with paracetamol and ibuprofen was immediately provided. Blood cultures, pus cultures from the eschars, as well as cultures from the vaginal exudate were obtained. Aggressive, broad-spectrum antibiotic treatment with meropenem, vancomycin, and clindamycin was initiated and the patient was transferred to the Department of Pediatric Surgery. 

The patient's clinical condition greatly improved in the first 48 hours of treatment, nevertheless three-week duration of antibiotic treatment and four extensive surgical debridements during that period were needed for the improvement of local lesions. Intraoperatively vast necrosis of skin, subcutaneous tissue, and muscle fascia were observed and tissue sections were obtained for histopathological and microbiological investigation. Urine diversion by the placement of a suprapubic catheter and fecal diversion by colostomy were also performed in order to protect the wounds from contamination.

Pus cultures grew *Staphylococcus aureus* and *Enterococcus faecium. Staphylococcus aureus* was also cultured in the vaginal exudate. No pathogens were isolated from blood and tissue specimens. Histology confirmed the diagnosis with typical findings for FG: vascular thrombosis, neutrophilic infiltration, subcutaneous tissue necrosis, and absence of primary muscle involvement. Features of hyperkeratosis, parakeratosis, papillomatosis, and hypergranulosis, findings consistent with HPV infection, were also detected, in the dermal area close to the necrotic lesion. Further laboratory investigation excluded underlying diseases that predispose to the development of necrotizing soft tissue infections and clinical entities that cause ulceration in the genital and perineal areas. These laboratory tests included autoantibodies specific for vasculitic and ulcerative autoimmune diseases, antibodies against viral and microbial pathogens, that is, CMV and EBV infection, HIV, HSV and other venereal infections, as wells as leishmanial and fungal diseases. Hematologic malignancies and immunodeficiencies were excluded by peripheral blood smear and immune phenotype tests.

The patient was discharged after the wounds had largely been healed, the colostomy and suprapubic catheter were removed, and the function of the bladder was restored. The remaining defects were allowed to heal with granulation tissue by secondary intension. In an 18-month followup, the deformity of the area had largely been restored (Figure 1).

## 3. Discussion

In the present patient, the diagnosis of FG was posed after the disease had progressed to the typical clinical picture of frank gangrene and was further supported by the laboratory investigation; the typical ultrasonographic findings, the common but unspecific finding of elevated infectious markers and the coagulopathy, a hallmark of childhood necrotizing soft tissue infections which is often helpful in the differentiation from nonnecrotizing infections [7]. Nevertheless, the patient had already been previously examined in the third and fourth days of illness but the existence of a necrotizing soft tissue infection had not been suspected, as apparently the necrotic lesions had not extended to the superficial layers of the skin by that time. Additionally, the initial presentation of FG can be misleading [6, 7]. Patients may present with fever, malaise, and nonspecific symptoms of infection without any complaints from the perineal area, although typically in the early stages the clinical hallmark of FG is severe local pain, disproportionate with physical findings [1]. Previously, cases of childhood necrotizing soft tissue infections escaped the diagnosis early in the course of the disease because they had presented with unspecific symptoms [7]. 

In contrast to adults where FG affects individuals with a predisposing condition, pediatric FG has been mostly observed in previously healthy children. However, an initiating event has been identified in most cases. Trauma, periurethral and perianal infections, and more rarely systemic diseases have been related to childhood FG [5]. Varicella infection has been associated with several cases of infant FG [8, 9]. In this prepubertal previously healthy girl, none of the previously described predisposing factors was detected, as underlying conditions and diseases that may present with perineal ulcers such as hematologic malignancies, vasculitic and ulcerative autoimmune diseases, and viral, microbial, and venereal infectious diseases [3, 10, 11] were excluded.

Most probably, in this case another common benign viral dermal infection, warts, was the sight of entry for pathogens that led to the development of FG, as warts were detected both by clinical examination and by histopathology close to the necrotic lesions of FG. Warts are the primary clinical manifestation of HPV infection. Genital warts in children may occur from incidental spread from cutaneous warts. A significant proportion of genital warts in children contain HPV types that are usually isolated from cutaneous warts. Nevertheless, all types of warts are very common among school-aged children and adolescents with a prevalence of 16% [12]. HPV lesions may be friable and become superinfected [13]. However, to our knowledge, the correlation of warts with the development of a necrotizing soft tissue infection has not been previously reported.

The course of FG in children is more benign, and antibiotic treatment with a more conservative surgical approach compared to that of adults has been described to lead to a favorable outcome [5]. Nonetheless, the optimal treatment of childhood FG has not been established by controlled trials. In recent reports of FG in infants and toddlers both antibiotic treatment and extensive surgical debridement were applied [6, 8]. Taking into consideration these data and also the fact that a late diagnosis may lead to an adverse outcome [1, 7], an aggressive approach was adopted for our patient. The applied antibiotic treatment provided coverage against the isolated pathogens: *Staphylococcus aureus*, a common cause of pediatric FG [5] and *Enterococcus faecium,* an opportunistic pathogen of FG [1]. Four extensive surgical debridements were needed for the removal of necrotic tissue, whereas fecal and urine diversion were undertaken to protect the wounds from contamination in order to accelerate recovery. Such interventions have been reported in childhood FG, mostly in patients where the site of infection was localized either in the rectum or in the urinary tract, but also in some patients solely for the local protection from contamination [5, 6, 9]. 

In conclusion, the diagnosis of FG is mostly clinical; in addition, in the childhood population otherwise benign infections may represent the initiating event for the development of this potentially fatal infection; therefore, the clinician must be aware of its signs and symptoms.
